# Cancer-related cognitive impairment in patients with newly diagnosed aggressive lymphoma undergoing standard chemotherapy: a longitudinal feasibility study

**DOI:** 10.1007/s00520-022-07153-9

**Published:** 2022-06-14

**Authors:** Priscilla Gates, Meinir Krishnasamy, Carlene Wilson, Eliza A. Hawkes, Vincent Doré, Yuliya Perchyonok, Christopher C. Rowe, Adam K. Walker, Janette L. Vardy, Michiel B. de Ruiter, Tania Cushion, Haryana M. Dhillon, Karla Gough

**Affiliations:** 1 Department of Clinical Haematology, Austin Health, Melbourne, Victoria Australia; 2 Department of Nursing, Faculty of Medicine, Dentistry & Health Sciences, The University of Melbourne, Melbourne, Victoria Australia; 3 Academic Nursing Unit, Peter MacCallum Cancer Centre, Melbourne, Victoria Australia; 4 Research and Education Nursing, Victorian Comprehensive Cancer Centre Alliance, Melbourne, Victoria Australia; 5 Olivia Newton-John Cancer Wellness and Research Centre, Austin Health, Melbourne, Victoria Australia; 6 School of Psychology and Public Health, LaTrobe University, Melbourne, Victoria Australia; 7 Faculty of Medicine, Dentistry & Health Sciences, The University of Melbourne, Melbourne, Victoria Australia; 8 Olivia Newton-John Cancer Research Institute (ONJCRI), Austin Health, Melbourne, Victoria Australia; 9 Department Molecular Imaging and Therapy, Austin Health, Melbourne, Victoria Australia; 10 The Australian E-Health Research Centre, CSIRO Health & Biosecurity, Melbourne, Victoria Australia; 11 Department Radiology, Austin Health, Melbourne, Victoria Australia; 12 Neuroscience Research Australia, Sydney, New South Wales Australia; 13 University of New South Wales, Sydney, New South Wales Australia; 14 Faculty of Medicine and Health, University of Sydney, Sydney, Australia; 15 Concord Cancer Centre, Concord Repatriation and General Hospital, Concord, New South Wales Australia; 16 Netherlands Cancer Institute, Amsterdam, The Netherlands; 17 Faculty of Science, School of Psychology, Centre for Medical Psychology& Evidence-Based Decision-Making, The University of Sydney, Sydney, New South Wales Australia; 18 Department of Health Services Research, Peter MacCallum Cancer Centre, Melbourne, Victoria Australia

**Keywords:** Feasibility study, Aggressive lymphoma, Cancer-related cognitive impairment

## Abstract

**Purpose:**

Cancer-related cognitive impairment (CRCI) is a recognised adverse consequence of cancer and its treatment. This study assessed the feasibility of collecting longitudinal data on cognition in patients with newly diagnosed, aggressive lymphoma undergoing standard therapy with curative intent via self-report, neuropsychological assessment, peripheral markers of inflammation, and neuroimaging. An exploration and description of patterns of cancer-related cognitive impairment over the course of treatment and recovery was also undertaken and will be reported separately.

**Methods:**

Eligible participants completed repeated measures of cognition including self-report and neuropsychological assessment, and correlates of cognition including blood cell–based inflammatory markers, and neuroimaging at three pre-specified timepoints, time 1 (T1) — pre-treatment (treatment naïve), time 2 (T2) — mid-treatment, and time 3 (T3) — 6 to 8 weeks post-completion of treatment.

**Results:**

30/33 eligible patients (91%, 95% CI: 76%, 97%) were recruited over 10 months. The recruitment rate was 3 patients/month (95% CI: 2.0, 4.3 patients/month). Reasons for declining included feeling overwhelmed and rapid treatment commencement. Mean age was 57 years (SD = 17 years) and 16/30 (53%) were male. Most patients (20/30, 67%) had diffuse large B cell lymphoma or Hodgkin lymphoma (4/30, 13%). The neuroimaging sub-study was optional, 11/30 participants (37%) were eligible to take part, and all agreed. The remaining 19 participants were ineligible as their diagnostic PET/CT scan was completed prior. Retention and compliance with all assessments were 89 to 100% at all timepoints. Only one participant was withdrawn due to disease progression.

**Conclusions:**

Findings from this study including excellent recruitment, retention, and compliance rates demonstrate it is feasible to longitudinally assess cognition in people with newly diagnosed aggressive lymphoma during their initial treatment and recovery to inform the development of future research to improve patient experiences and cognitive outcomes.

Trial registration.

Australian New Zealand Clinical Trials Registry ACTRN12619001649101.

**Supplementary Information:**

The online version contains supplementary material available at 10.1007/s00520-022-07153-9.

## Introduction

Cancer-related cognitive impairment (CRCI) is a highly distressing and disabling side effect commonly reported by cancer patients [1, 2]. The incidence varies, but studies of patients with solid tumours suggest that up to 70% of patients receiving treatment self-report some degree of cognitive impairment [1, 2]. The cognitive domains most commonly affected are memory, concentration, information processing, speed, and executive function [1]. For some patients, cognitive impairment may be transient, but for a subgroup, these symptoms can be long-lasting and have a major impact on quality of life and ability to function [3].

Lymphoma is the 6th most common cancer in adults and is the most common cancer in young people aged 15–29 years in Australia [4]. Aggressive lymphomas including Hodgkin lymphoma (HL) and non-Hodgkin lymphoma (NHL), such as diffuse large B-cell lymphoma (DLBCL), are potentially curable cancers. Current treatments, consisting of combination chemotherapy with or without radiotherapy, provide a 5-year progression-free survival (PFS) of 65–92% and potential cure in around 50% of patients [5]. This, coupled with a favourable prognosis, has resulted in a growing population of survivors of aggressive lymphoma, who are at risk of side effects from the long-term toxicity associated with the treatments received [6].

Persistent changes in cognitive function are frequently reported by lymphoma survivors [7–9]. The majority of CRCI studies have been performed in women with breast cancer, whilst several small-size studies incorporated patients with other solid tumours [10–12]. This population is generally older than people with aggressive lymphoma; however, they form the target population in the majority of CRCI studies. In young adults, with a longer life expectancy, impaired cognition may have a dramatic impact on their quality of life, affecting their working and learning capacities and multiple aspects of their social life. Despite such widespread implications, the feasibility of collecting longitudinal data on cognition in patients with aggressive lymphoma during treatment has not been explored.

The International Cognition and Cancer Task Force (ICCTF), formed with the goal of improving understanding of the impact cancer and its treatments have on cognition, recommends comprehensive, longitudinal neuropsychological assessment as the gold standard for measuring cognitive function [13]. Emerging evidence suggests that blood cell inflammatory markers may serve as a valuable prognostic indicator of cognitive impairment and susceptibility among cancer patients [14, 15], and neuroimaging studies have demonstrated cerebral structural changes associated with chemotherapy [16–19]. Yet, few studies have assessed cognition in patients pre- and post-treatment [16–18], and there are limited data of cognitive changes in patients with haematological malignancies [20–22]. Given the need for urgent comprehensive diagnostic work-up and rapid commencement of chemotherapy among patients with aggressive lymphoma, establishing the feasibility of longitudinal, and particularly pre-treatment neuropsychological assessment, is an important goal, if the recommendations of the ICCTF are to be realised. To date, a longitudinal exploration of the pattern of CRCI over the course of treatment and recovery has not been described in this population.

Given the limited data on the feasibility of comprehensively assessing cognition in patients with non-CNS aggressive lymphoma before, during, and after treatment, it was important to explore this issue before embarking on a large-scale study to comprehensively describe the cognitive outcomes and trajectory of this patient cohort. We aimed to (1) assess the feasibility of collecting longitudinal data on cognition using self-report measures and objective neuropsychological tests in people with newly diagnosed aggressive lymphoma undergoing standard therapy with curative intent in a research setting, and (2) explore and describe patterns of CRCI in the population of interest as measured by self-report measures, neuropsychological tests, blood cell–based inflammatory markers, and neuroimaging. Here, we report findings relevant to the feasibility aim.

## Methods

### Study design and setting

**Fig. 1:**
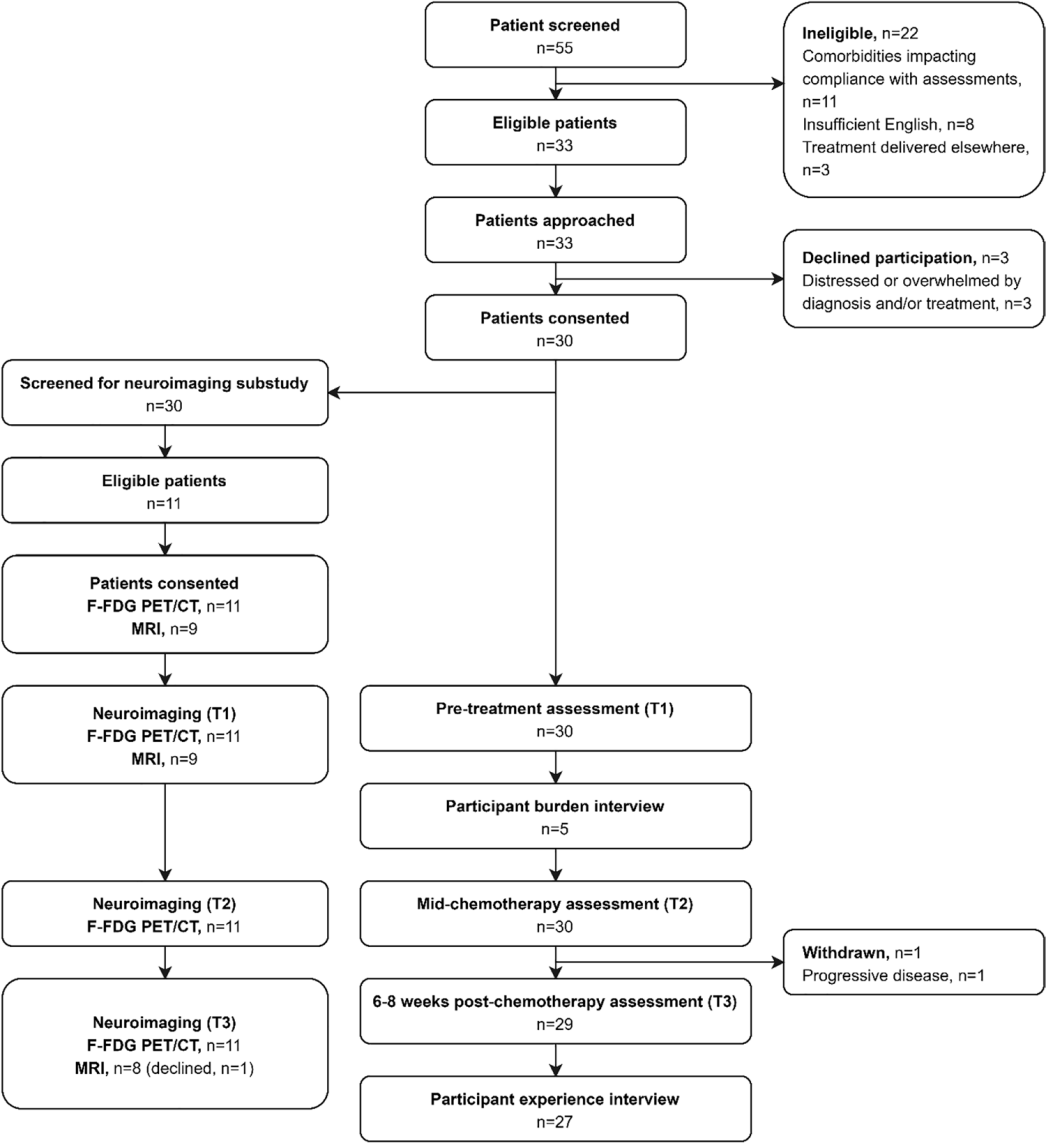
Study schema

This single-site, single-arm, longitudinal feasibility study was conducted in a specialised haematology department of a comprehensive cancer centre in a large acute hospital in metropolitan Melbourne, Australia. The published protocol provides a detailed description of study methods and procedures [23]. The study schema is presented in Fig. 1.

### Participants

Participants were 18 years or older with newly diagnosed aggressive lymphoma; scheduled to undergo standard combination chemotherapy with curative intent; able to read and comprehend English; and had a documented ECOG Performance Status 0 to 2 [24]. Patients were not eligible if they had any of the following: lymphomatous CNS involvement, prior or planned cranial radiation therapy, a life expectancy less than 12 months, any medical condition that might compromise adherence or lead to prolonged hospitalisation, a documented history of past or current substance misuse, or poorly controlled psychiatric illness.

### Recruitment procedures

Eligible patients were approached and invited to participate by the study nurse [23]. A copy of the participant information statement and consent form (PICF) was provided, then written informed consent obtained for self-report measures, neuropsychological tests, blood cell–based inflammatory markers, and screening for eligibility for neuroimaging sub-study.

### Data collection procedures for all study data

Consenting participants underwent comprehensive assessments at three pre-specified times: pre-chemotherapy (T1), mid-chemotherapy (post cycle 2) (T2), and 6–8 weeks post-chemotherapy (T3) with the study nurse (Fig. 1). Neuropsychological tests to assess relevant cognitive domains include learning and memory on the Hopkins Verbal Learning Test-Revised (HVLT-R), verbal written fluency on the Controlled Oral Word Association (COWA), executive function on the Stroop Colour and Word, and Trail Making Test Part B, speed of information processing on the Trail Making Test Part A, and attention/working memory on the Digit Span (WAIS-R). Patient-reported outcome measures included the EORTC Quality of Life Questionnaire Cognitive functioning scale (QLQ-C30 CF), the Functional Assessment of Cancer Therapy-General (FACT-G) and -Cognitive Function (FACT-Cog), Cognitive Failures Questionnaire (CFQ), Functional Assessment of Chronic Illness Therapy-Fatigue (FACIT-F), and PROMIS Emotional Distress-Depression 8b and -Anxiety 7a short forms. A detailed description of each measure is provided in the protocol paper [23].

Laboratory tests including full blood examination (FBE) were collected as part of standard care. These parameters were used to calculate blood cell–based inflammatory markers including the neutrophil to lymphocyte ratio (NLR), platelet to lymphocyte ratio (PLR), and systemic immune-inflammation index (SII). Neuroimaging (^18^F-FDG PET/CT brain acquisition study and MRI scan) formed part of an optional sub-study and the brain MRI sub-study occurred at two timepoints only. Patients who had already had their standard of care diagnostic PET scans before attending the lymphoma service were not eligible to participate in the sub-study. Interview data were collected to explore participant burden and a qualitative sub-study explored motivation to participate. A synopsis of study findings is reported here for completeness; the findings of the qualitative sub-study are reported in full elsewhere [25] (refer to Fig. 1).

### Analysis

#### Feasibility outcomes

The main feasibility outcomes were to estimate the recruitment rate (i.e. the number of patients recruited per month), the retention rate, compliance with study measures, and the proportions of patients who were willing to have ^18^F-FDG PET/CT and MRI scans, as well as to assess the acceptability of subjective and objective study measures and the practicability of blood collection. Recruitment data were summarised using a rate and 95% CI estimated using the Poisson distribution. Retention, compliance with assessments, and consent and assessment context data were summarised using counts and percentages. The acceptability of study measures was explored via a face-to-face participant burden interview. Findings were analysed using a summarising content analysis. The consent rate (i.e. eligible patients approached and consented) was summarised using a proportion and 95% confidence intervals (CI) estimated using the Wilson method. The context of consent and study assessments was summarised using counts and percentages.

#### Participant characteristics and study measures

Study measures were scored according to author guidelines. Descriptive statistics (counts and percentages; means, standard deviations, and/or 95% CI; medians and interquartile ranges; and ranges, as appropriate) were used to summarise participant characteristics, missing data, pre-chemotherapy study measure scores, and score changes at follow-up assessments (T2 and T3) from pre-chemotherapy, as well as pre-chemotherapy blood cell–based inflammatory markers and changes in these markers at follow-up assessments (T2 and T3) from pre-chemotherapy. Confidence intervals for the latter were bootstrapped (10,000 replications) due to the small sample and observable skew. Kazis et al. [26] effect size estimates were calculated to characterise the sizes of changes from pre-chemotherapy. Linear mixed models were used to assess the overall pattern of change in self-report measures and neuropsychological tests. All models included a fixed effect for time and random participant effect. All analysis was performed in R (version 3.6.1) [27], using ‘epitools’ [28], ‘binom’ [29], ‘wBoot’ [30], and ‘lme4’ [31].

## Results

### Study profile

**Fig. 2:**
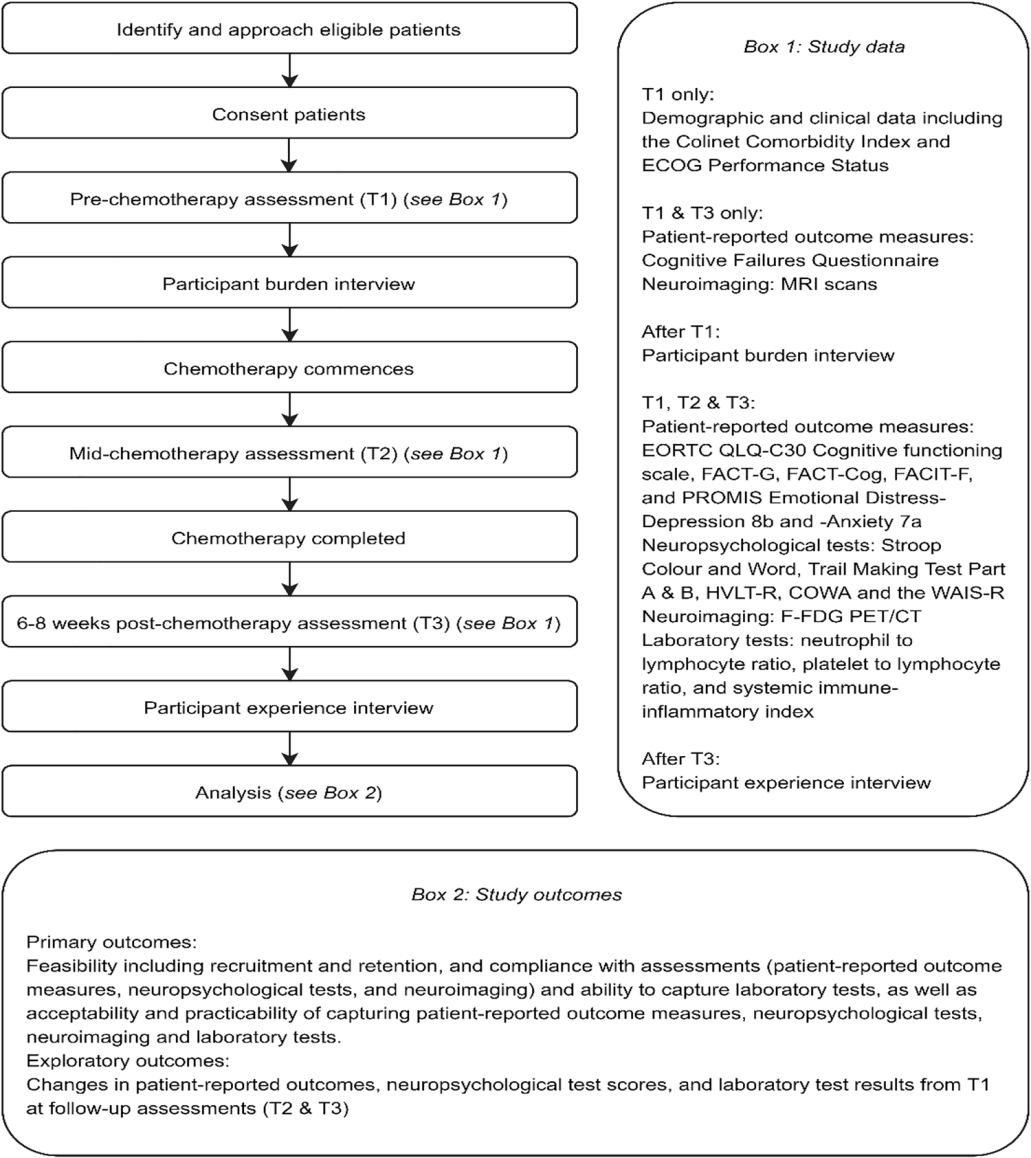
Participant flow diagram

Fifty-five patients with newly diagnosed aggressive lymphoma were screened for eligibility between 26 November 2019 and 01 September 2020. Twenty-two patients were ineligible. Reasons for ineligibility are summarised in Fig. 2. The main reason for ineligibility was medical conditions potentially compromising adherence or leading to prolonged hospitalisation *n* = 11, and this included co-existing illnesses or behavioural issues, cancer-related pain, and cancer-related nausea requiring hospitalisation. Thirty-three patients met eligibility criteria and were approached and 30 of 33 (91%, 95% CI: 76%, 97%) consented to participate. PET/CT brain scans were obtained for 11 of 30 (37%) participants; the remaining participants had had their diagnostic PET scans before attending the lymphoma service.

Characteristics of the study sample pre-chemotherapy are summarised in Table 1. The median age was 57 years (range 18 to 78 years) and 16/30 (53%) participants were male. Most participants were diagnosed with DLBCL (*n* = 20, 66%) or HL (*n* = 4; 14%). All participants diagnosed with aggressive lymphoma received combination chemotherapy as per standard of care.

**Table 1:** Participant characteristics (*n* = 30)

Characteristics	*n*	%
Age at enrolment, in years		
Mean (SD)	57 (17)	
Median (IQR)	61 (50 to 69)	
Range	18 to 78	
Sex		
Male	16	53
Female	14	47
Country of birth		
Australia	23	77
Other	7	23
Language spoke at home		
English	28	93
Other	2	7
Marital status		
Married/de facto	21	70
Separated/divorced	2	7
Single	6	20
Widowed	1	3
Work status prior to diagnosis		
Full time	12	40
Home care duties	1	3
Part time	3	10
Retired	11	37
Sickness benefits	1	3
Unemployed	1	3
Student	1	3
Years of formal education		
Mean (SD)	13 (2)	
Median (IQR)	13 (12 to 14)	
Range	7 to 18	
ECOG performance status		
0	21	70
1	9	30
Colinet comorbidity score		
Median (IQR)	0 (0 to 1)	
Range	0 to 9	
Active psychiatric/medication affecting cognition		
No	27	90
Yes	3	10
Been treated for depression/anxiety/psychiatric/neurological condition		
No	22	73
Yes	8	27
Anxiety	2	
Chronic schizophrenia	1	
Depression	4	
Panic attacks	1	
Diagnosis		
DLBCL	20	67
Grade 3B follicular lymphoma	1	3
HL	4	13
Mantle cell lymphoma	1	3
T-cell lymphoma	3	10
Primary mediastinal B-cell lymphoma	1	3
Chemotherapy regimen		
ABVD × 6	3	10
CHOP × 6	2	7
Esc-BEACOPP × 4	1	3
Mini R-CHOP × 6	2	7
R-CHOP × 2	1	3
R-CHOP × 3	2	7
R-CHOP × 4	3	10
R-CHOP × 6	10	33
R-CHOP & HD MTX × 2	1	3
R-CHOP & R × 2	4	13
R-CHOP/R-DHAP × 3	1	3
Length of chemotherapy treatment, in days		
Mean (SD)	102 (34)	
Median (IQR)	105 (105 to 114)	
Range	21 to 116	

*ABVD*, adriamycin, bleomycin, vinblastine, dacarbazine; *R-CHOP*, rituximab, cyclophosphamide, doxorubicin, vincristine, prednisolone; *Esc-BEACOPP*, bleomycin, etoposide, doxorubicin, cyclophosphamide, vincristine, procarbazine, prednisolone; *HD* *MTX*, high-dose methotrexate; *R-DHAP*, rituximab, dexamethasone, cytarabine, cisplatin

### Feasibility outcomes

#### Recruitment rate

The recruitment rate was 3 patients/month (95% CI: 2.0, 4.3 patients/month).

#### Retention rate

Retention at follow-up was excellent (Fig. 2); only one participant was withdrawn from the study between T2 and T3 as a consequence of disease progression.

#### Feasibility of consent and timing of diagnosis

The median time between patient informed of diagnosis and consent was 2 days (inter-quartile range: 0 to 7 days; range: 0 to 33 days). The median time between patient informed of diagnosis and pre-chemotherapy assessment was 7 days (inter-quartile range: 2 to 14 days; range: 0 to 49 days). The median time between patient informed of diagnosis and treatment commencement was 12 days (inter-quartile range: 7 to 17 days; range: 0 to 99 days).

#### Consent and assessment context

The context of consent and study assessments is summarised in Table 2. Most patients (20/30) were approached and invited to participate whilst attending the lymphoma outpatient clinic situated in the cancer centre; seven were consented as inpatients. Six participants completed their pre-chemotherapy neuropsychological assessment in the day oncology unit prior to their chemotherapy infusion; six were in lymphoma outpatient clinic and six were inpatients on the haematology ward. Some participants (13/30) completed their mid-chemotherapy neuropsychological assessment on the day their PET scan was scheduled; ten completed this assessment in the day oncology unit prior to the commencement of their third cycle of chemotherapy. The majority of participants (19/30) completed their 6–8 weeks post-chemotherapy neuropsychological assessment on the day their PET or MRI scan was scheduled.

**Table 2:** Consent and assessment context

Context	*n*	%
Consent, *n* = 30		
Day of chemotherapy	2	7
Day of chemotherapy education	1	3
Lymphoma clinic	20	67
Inpatient haematology	5	17
Inpatient other	2	7
Pre-chemotherapy assessment, *n* = 30		
Day of chemotherapy commence	6	20
Day of chemotherapy education	5	17
Inpatient	6	20
Lymphoma clinic	6	20
Post-PET scan	2	7
Post-MRI scan	3	10
Stand-alone appointment	2	7
Mid-chemotherapy assessment, *n* = 30		
Day of chemotherapy	10	33
Inpatient	1	3
Pre-PET scan	7	23
Lymphoma clinic	5	17
Post-PET scan	6	20
Stand-alone appointment	1	3
6–8 weeks post-chemotherapy assessment, *n* = 29		
Lymphoma clinic	4	14
Pre-MRI scan	1	3
Pre-PET scan	9	31
Post-PICC line removal	3	10
Post-PET scan	9	31
Stand-alone appointment	3	10

*PET* *scan*, positron emission tomography scan; *MRI* *scan*, magnetic resonance imaging scan; *PICC* *line*, peripherally inserted central catheter

#### Compliance with study measures

Compliance with study measures was high at all timepoints. For patient-reported outcome measures, compliance was 90 to 100% at T1, T2, and T3 (Table 3). For neuropsychological assessment, compliance was 100% at T1, T2, and T3 for all active participants (Table 4). Laboratory tests were available for all active participants at T1, T2, and T3.

**Table 3:** Descriptive statistics for patient-reported outcome measures, pre-chemotherapy scores, and mean changes at follow-up assessments

Measure	Pre-chemotherapy			Mid-chemotherapy					6–8 weeks post-chemotherapy				
	*n*	*M*	*SD*	*n*	*M*	*SD*	M chg (95% CI)	ES	*n*	*M*	*SD*	M chg (95% CI)	ES
EORTC QLQ-C30 Cognitive functioning													
Subscale score	30	84.4	19.5	29	81.6	20.6	− 2.3 (− 8.6, 4.0)	0.12	29	78.2	18.4	− 6.9 (− 14.8, 1.0)	0.35
FACT-Cog													
Perceived cognitive impairment	30	60.8	11.9	30	56.2	16.2	− 4.6 (− 8.3, − 0.9)	0.39	29	57.1	15.3	− 4.2 (− 8.1, − 0.2)	0.35
Impact of perceived impairment on QOL	30	11.3	4.7	29	11.9	4.4	0.8 (− 0.7, 2.2)	0.16	29	11.6	4.4	0.5 (− 1.4, 2.4)	0.10
Perceived cognitive abilities	30	21.7	6.2	29	18.7	8.4	− 3.0 (− 6.0. 0.0)	0.47	29	19.4	7.1	− 2.5 (− 4.4, − 0.5)	0.39
Cognitive Failures Questionnaire													
Forgetfulness	30	9.6	4.9						29	10.3	5.2	0.7 (− 0.8, 2.2)	0.14
Distractibility	30	9.2	5.1						29	9.5	5.5	0.5 (− 1.1, 2.1)	0.09
False triggering	30	5.8	4.1						29	7.1	4.9	1.3 (− 0.3, 3.0)	0.33
FACT-G													
Physical wellbeing	29	21.2	6.3	30	19.4	7.2	− 1.8 (− 4.8, 1.1)	0.29	29	20.6	5.5	− 1.3 (− 3.8, 1.2)	0.24
Social wellbeing	29	23.2	4.9	29	23.4	5.0	− 0.2 (− 1.1, 0.8)	0.03	28	23.4	5.2	0.1 (− 1.1, 1.3)	0.02
Emotional wellbeing	28	16.4	5.1	30	18.8	4.1	2.4 ( 0.5, 4.2)	0.47	29	19.3	3.8	2.9 (1.3, 4.4)	0.55
Functional wellbeing	28	19.5	6.2	30	18.1	6.0	− 1.6 (− 3.6, 0.4)	0.25	29	18.1	5.9	− 1.4 (− 3.2, 0.4)	0.24
Total score	28	80.2	15.6	29	79.6	16.0	− 1.1 (− 6.5, 4.3)	0.07	28	81.4	15.2	0.3 (− 4.6, 5.1)	0.02
FACIT-F													
Total score	27	37.3	13.3	30	33.8	12.4	− 3.7 (− 7.8, 0.4)	0.28	29	32.6	11.5	− 5.6 (− 9.7, − 1.6)	0.48
PROMIS Emotional Distress													
Depression 8b	28	51.5	8.3	27	50.3	10.4	− 1.7 (− 4.7, 1.3)	0.20	27	49.0	9.1	− 2.3 (− 5.2, 0.6)	0.27
Anxiety 7a	28	55.8	9.9	29	49.1	10.3	− 6.6 (− 10.1, − 3.2)	0.67	27	48.2	9.4	− 7.4 (− 10.7, − 4.0)	0.72

*M* *chg*, mean change at follow-up assessments from baseline (interpretation of M chg for measures described below); *CI*, confidence interval; *ES*, Kazis effect size (interpretation of mean changes: 0.2, small; 0.5, medium; and 0.8, large)

Interpretation of M chg: for the EORTC Cognitive functioning subscale score, FACT-G domain and total scores, and FACIT-F total score, a positive M chg reflects improvement, and a negative M chg reflects deterioration; for FACT-Cog scales, Cognitive Failures Questionnaire scales, and PROMIS Emotional Distress measures, a positive M chg reflects deterioration, and a negative M chg reflects improvement

**Table 4:** Descriptive statistics for neuropsychological tests, pre-chemotherapy scores, and mean changes at follow-up assessments

Measure	Pre-chemotherapy			Mid-chemotherapy					6–8 weeks post-chemotherapy				
	*n*	*M*	*SD*	*n*	*M*	*SD*	M diff (95% CI)	ES	*n*	*M*	*SD*	M diff (95% CI)	ES
Stroop Colour and Word Test													
Colour	30	43.8	9.1	30	43.0	10.7	− 0.8 (− 2.9, 1.2)	0.09	29	41.7	9.0	− 2.2 (− 4.3, − 0.1)	0.23
Word	30	43.0	10.9	30	42.9	10.1	− 0.2 (− 3.1, 2.8)	0.02	29	42.6	9.8	− 0.7 (− 4.2, 2.8)	0.07
Word/colour	30	48.2	10.3	30	48.6	9.3	0.4 (− 2.3, 3.1)	0.04	29	48.2	8.6	0.1 (− 2.9, 3.1)	0.01
Inference colour/word	30	46.8	8.9	30	48.2	8.8	1.4 (− 1.3, 4.1)	0.16	29	48.1	8.2	1.7 (− 1.0, 4.4)	0.19
Trail Making Test													
A score	30	45.8	9.6	30	47.6	8.8	1.8 (− 0.7, 4.3)	0.19	29	48.6	9.6	3.1 (− 0.3, 6.5)	0.32
B score	30	46.6	13.8	30	49.0	14.1	2.4 (− 0.4, 5.2)	0.17	29	47.3	14.1	0.7 (− 4.2, 5.6)	0.05
Hopkins Verbal Learning Test													
Total recall	30	40.8	10.0	30	44.9	9.6	4.1 (0.0, 8.2)	0.40	29	45.9	11.8	5.2 (1.1, 9.3)	0.51
Delayed recall	30	38.1	12.4	30	41.7	12.7	3.5 (− 1.8, 8.9)	0.28	29	41.6	12.5	3.6 (− 1.5, 8.7)	0.28
Retention	30	41.8	13.6	30	43.2	14.0	1.4 (− 5.8, 8.6)	0.10	29	41.9	12.2	− 0.1 (− 6.1, 5.9)	0.01
Recognition/discrimination	30	46.7	11.6	30	39.3	14.9	− 7.4 (− 12.6, − 2.2)	0.64	29	47.9	10.3	1.5 (− 2.5, 5.4)	0.13
Controlled Oral Word Association Test													
Total letter fluency	30	42.3	11.9	30	48.2	11.4	5.9 ( 2.3, 9.5)	0.50	29	46.0	12.2	3.4 (− 0.2, 7.1)	0.29
Category fluency	30	46.5	10.5	30	45.8	12.4	− 0.7 (− 3.6, 2.3)	0.06	29	46.5	10.6	0.0 (− 2.9. 2.8)	0.00
Total written fluency	30	44.5	11.7	30	46.7	14.4	2.2 (− 0.7, 5.0)	0.19	29	48.7	15.2	4.1 (1.4, 6.8)	0.34
Digit Span Wechsler Adult Intelligence Scale													
Digit span total	30	48.2	8.1	30	50.1	9.9	1.8 (− 0.6, 4.3)	0.23	29	49.5	9.1	1.1 (− 1.1, 3.4)	0.14

*M* *chg*, mean change at follow-up assessments from baseline (interpretation of M chg for measures described below); *CI*, confidence interval; *ES*, Kazis effect size (interpretation of mean changes: 0.2, small; 0.5, medium; and 0.8, large)

Interpretation of M chg: for all measures, a positive M chg reflects an improvement in cognitive performance, and a negative M chg reflects a deterioration in cognitive performance

All 30 participants were screened for the neuroimaging sub-study. Nineteen participants were ineligible as the diagnostic standard of care whole-body PET/CT scan was completed prior to attending the lymphoma service. A total of eleven participants met the eligibility criteria and were invited to participate. All (11; 100%) agreed to participate; however, two participants declined MRI brain study due to claustrophobia and anxiety from previous MRI scans.

Compliance with the PET scans was 100% (11 of 11) at T1, T2, and T3 for eligible participants. Compliance with the MRI scans was 100% (9 of 9) at T1 and 89% (8 of 9) at T3 (Fig. 2) for consenting participants. This is despite some participants describing the additional neuroimaging requirements as time consuming, uncomfortable, and anxiety provoking. One participant declined the final MRI due to distress related to disease progression. Disease progression was identified 1 week after other study measures were completed.

Several feasibility issues were identified in collection of the neuroimaging data. The MRI study included small sample size and was only performed at two timepoints. For the PET scans, there were large time delays between scan acquisition and FDG tracer injection in five patients, variability in the scanner used, and variability in days between T1 and follow-up imaging. Therefore, we have addressed and reported feasibility objectives only.

#### Acceptability of subjective and objective study measures

To explore acceptability of the neuropsychological assessment and self-report measures in a population for whom there is no reported data, the first five participants enrolled completed a face-to-face burden interview 1 week after completion of the baseline assessments. Two participants thought components of the self-report measures were repetitive; two found individual neuropsychological tests difficult; one found the assessment tiring; and another felt time to complete was longer than expected. Nevertheless, none of the participants recommended changes to the assessment schedule [32].

### Patient-reported outcome measures and neuropsychological assessments

Descriptive statistics for pre-chemotherapy patient-reported outcome measures and neuropsychological test scores, as well as changes from pre-chemotherapy at follow-up assessments (T2 and T3) are provided in Tables 3 and 4, respectively. Linear mixed model results are provided in online supplemental Appendices A and B for completeness.

Small- to medium-sized changes were observed on a number of patient-reported outcome measure scale/total scores (Table 3); for the most part, changes reflected a deterioration in relevant domains at follow-up, apart from changes in anxiety and depression, which indicated improvement at follow-up assessments.

Small- to medium-sized changes were observed on some neuropsychological test scale/total scores (Table 4); for the most part, changes suggested improvement at follow-up assessments.

### Blood cell–based inflammatory markers

Descriptive statistics for pre-chemotherapy blood cell–based inflammatory markers and changes in these markers at follow-up assessments are provided in online supplemental Appendix C for completeness*.* Small- to medium-sized changes were observed on the NLR and PLR. In a study conducted in 166 survivors of breast cancer, higher blood cell inflammatory markers reflected lower cognitive performance compared to healthy controls [15].

### Motivation for participation

Four themes were generated from the qualitative interview data describing participants motivation for sustained participation in this study, at the time of heightened stress related to a new diagnosis of aggressive lymphoma: (1) ease of participation; (2) personal values that impact attitude to participation; (3) desire to engage in self-help; and (4) appreciation of additional support. Data and insights from the qualitative sub-study have been published [25].

## Discussion

Our study suggests it is feasible to complete a comprehensive assessment of cognitive outcomes in 30 patients with newly diagnosed aggressive lymphoma over the course of treatment and recovery.

Despite the distressing, challenging, and stressful nature of the lymphoma diagnosis, recruitment to our study and completion of baseline assessments exceeded our expectations. Thirty people with newly diagnosed aggressive lymphoma were recruited over a 10-month period, and only three declined participation. Participants may have been motivated to take part to help others. Results describing motivation and reasons for sustained participation have been published previously [25].

People willing to participate in the study were often consented within days of diagnosis. Thirteen (43%) were consented on day of being informed of diagnosis. Consent was obtained in a variety of clinical settings, with seven (23%) participants inpatients, five in the dedicated haematology ward, and two in general surgical wards. The median time between being informed of diagnosis and pre-chemotherapy neuropsychological assessment was 7 days (range 0 to 49), with 4 (13%) participants informed of diagnosis, consenting to the study and assessed on the same day. The environment for collection of neuropsychological data was flexible and included a variety of clinical settings (e.g. day oncology unit, outpatient clinic, or inpatient unit). Management of potential disruption by clinical staff in the inpatient setting was minimised by placing a ‘do not disturb’ sign on door. The median time between being informed of diagnosis and date of treatment commencement was 12 days (range 0 to 99); with one (3%) participant being informed of diagnoses, consented, assessed, and commencing treatment on the same day.

Retention and compliance with all measures were excellent, despite literature reporting challenges with recruitment of patients recently been diagnosed with cancer [33]. Other studies have reported that retention of participants to longitudinal studies can be challenging [34–36], and attrition is often attributed to poor study design [35]. A recent study by Janelsins et al. (2021) assessed longitudinal changes in cognition in patients with lymphoma before and after chemotherapy; however, they did not include a mid-treatment assessment. They experienced significant attrition throughout the study; retention immediately post-chemotherapy was 86% and 72% 6 months post-chemotherapy, with losses to follow-up being the main reason for attrition [9]. This may have been multifactorial and exacerbated by there being no mid-treatment assessment, potentially causing lack of interest or motivation for participants to stay engaged.

Success with recruitment and compliance with assessments in the current study was likely due to flexibility in testing time and location, reflecting the availability of the study nurse across all clinical settings. In particular, this flexibility enabled study assessments to take place at times convenient to the participants, usually when they were already at the hospital, thereby reducing any additional travel demands. Importantly, participants were approached at the hospital by the study nurse, possibly contributing to the high recruitment rate. The study nurse may have been seen as a trustworthy credible member of the team [37]. Whilst not involved in clinical care at the time of diagnosis, the study nurse responsible for recruiting patients and conducting assessments was an experienced haematology nurse and a clinician nurse-researcher [23]. As such she was in a privileged position, being a long-standing staff member in the clinical setting, which may have strengthened the clinical team’s motivation to identify and refer patients to the study [38]. Additionally, potential participants likely appreciated the study nurse’s clinical expertise, and this may have improved trust among patients, encouraging participation [39–41]. These observations highlight the potential benefit of clinician involvement in data collection [42], strengthening capacity for clinical research, notwithstanding the related ethical concerns of the study nurse-participants relationship grounded in trust but open to participant coercion.

Exploratory findings based on analysis of responses to neuropsychological tests and patient-reported outcome measures demonstrated estimates of change in cognitive function based on neuropsychological tests provided evidence of improvement in verbal fluency and memory. Conversely, estimates of change in cognitive function based on self-report measures provided evidence of deterioration in perceived cognitive impairment and abilities. A greater description of exploratory findings is currently under preparation.

Interviews were conducted to examine the acceptability of the study protocol. A participant burden interview was undertaken with the first five participants, and no changes to the assessment schedule were recommended by any participant [32]. Feasibility of the study was later confirmed via a separate qualitative sub-study that explored the motivations for initial and ongoing participation at a time of heightened stress related to a new diagnosis of aggressive lymphoma and the rapid commencement of treatment. Ease of participation; personal values that impact attitude to participation; desire to engage in self-help; and appreciation of additional support motivated ongoing participation [25].

Despite all eligible participants agreeing to participate in the neuroimaging sub-study, only 11 of 30 (37%) were deemed eligible as diagnostic PET/CT scans had been acquired prior to attending the lymphoma service for the other patients. Our results speak to the difficulty of capturing baseline neuroimaging assessments in all patients; however, in those eligible for the sub-study, it was feasible. We found challenges with collecting longitudinal research quality PET/CT brain acquisition studies, which require constraints on scan time acquisition and camera availability, to assess any biomarker change over time, was difficult to implement in a clinical setting. Notwithstanding excellent compliance among those who were eligible, some participants found the brain scans to be challenging, arduous, and time consuming, and thus these data should only be collected in future studies if critical, given reports of participant burden.

This study has limitations that impact the generalisability of results. The sample size was small, and participants were recruited from a single institution. Furthermore, despite the high proportion of patients consenting to participate, the involvement of a haematology nurse specialist may limit replicability in other centres. Whilst longitudinal, our study assessments are limited to 6 months post-treatment completion. This reduces the capacity to explore and describe patterns of CRCI with repeat assessment in patients with aggressive lymphoma long into recovery, which is important given the potential long-term survivorship.

The excellent compliance with laboratory tests may be unsurprising given they were standard of care. All participants in our study consented to access to routine laboratory data. To ensure access in future studies, availability and consent to use of this data must be considered. There were several challenges identified in the neuroimaging sub-study which were detailed in the ‘Results’ section. High levels of ineligibility may impact future studies if those who were ineligible were systematically different, which may introduce bias in future longitudinal studies of the same design. We recommend strong collaboration with the PET imaging centre, and longer imaging slots for each participant. However, this may increase costs associated with imaging and potentially place more demands on participants, who are already under considerable stress. Future studies should aim to recruit a larger patient cohort from across multiple institutions and should include patients from different cancer groups.

## Implications for practice

Our study suggests that it is feasible to complete a comprehensive assessment of cognitive outcomes in patients with newly diagnosed aggressive lymphoma over the course of treatment and recovery. Our findings can inform the development of future studies and can be used to implement and evaluate formal cognitive testing as part of standard of care.

Twenty-two patients were ineligible for the study, of which half was due to a medical condition that may compromise adherence or lead to prolonged hospitalisation. The relevance and value excluding people from cognitive assessments as part of intervention studies must be carefully considered in the future. If such a high proportion of people were excluded, the relevance of assessment and interventions for routine practice would be questionable.

Success with recruitment, retention, and compliance with all study measures throughout demonstrates nurse-led assessments are feasible. This approach in research settings offers a more practical, time-effective, and cost-effective way of assessing cognitive function than other options. Additionally, in routine practice, nurses could more easily screen for cognitive changes to support referral to neuropsychologists for detailed assessment and support using a stepped care approach.

Despite all eligible participants agreeing to participate in the neuroimaging sub-study, only 11 were eligible. For those ineligible, diagnostic PET/CT scans had been acquired prior to attending the lymphoma service. Our results speak to the difficulty of capturing standardised baseline neuroimaging assessments in all patients as part of research studies. It also has implications for appropriate models of care required to translate neuroimaging as part of standard lymphoma care.

## Conclusion

Findings from this study demonstrate that it is feasible to assess cognitive status in people with newly diagnosed aggressive lymphoma during their initial treatment. Our recruitment to the study despite being a time of heightened stress was excellent. Compliance and retention with all study measures, including patient-reported outcome measures, neuropsychological assessments, laboratory tests, and neuroimaging in eligible participants, were very high at all timepoints. Our results suggest that longitudinal assessment of cognitive function in patients during treatment and recovery is acceptable to the patients and therefore feasible. However, some of the data capture strategies were onerous and research quality PET scans were difficult to acquire in clinical settings and require careful consideration before including in future studies. We recommend future large-scale studies should be undertaken to comprehensively describe cognitive outcomes and trajectory including larger patient groups within other cancer groups.

## Supplementary Information

Below is the link to the electronic supplementary material.

Supplementary file1 (DOCX 30 KB)

## Data Availability

De-identified data supporting the findings of this study are available from the corresponding author upon request.

## Code availability

NA.
