# Supplementary material for: Cancer-related cognitive impairment in patients with newly diagnosed aggressive lymphoma undergoing standard chemotherapy: a longitudinal feasibility study
Source: Support Care Cancer. 2022 Jun 14;30(9):7731–43. doi: 10.1007/s00520-022-07153-9 (PMC9385796; doi:10.1007/s00520-022-07153-9)
Supplement: Supplementary file 1 — Supplementary file1 (DOCX 30 KB) [file 520_2022_7153_MOESM1_ESM.docx]

# Supplemental Appendices

### Appendix A: Linear mixed model results for patient-reported outcome measures

| Measure/(sub)scale | Pre-chemotherapy | | Mid-chemotherapy chg | | 6-8 weeks post-chemotherapy chg | | *p*-value |
| --- | --- | --- | --- | --- | --- | --- | --- |
|  | est. | *se* | est. | 95% CI | est. | 95% CI |  |
| EORTC Cognitive Functioning scale |  |  |  |  |  |  |  |
| Total score | 84.4 | 3.5 | -2.8 | (-10.1, 4.5) | -6.6 | (-14.0, 0.7) | 0.20 |
| FACT-Cog |  |  |  |  |  |  |  |
| Perceived cognitive impairment | 60.8 | 2.6 | -4.6 | (-8.4, -0.8) | -4.4 | (-8.2, -0.5) | 0.03 |
| Impact of perceived impairment on QOL | 11.3 | 0.8 | 0.6 | (-1.1, 2.3) | 0.3 | (-1.4, 2.0) | 0.80 |
| Perceived cognitive abilities | 21.7 | 1.3 | -3.0 | (-5.4, -0.6) | -2.6 | (-5.0, -0.2) | 0.03 |
| Cognitive Failures Questionnaire |  |  |  |  |  |  |  |
| Cog Fails Forgetfulness | 9.6 | 0.9 |  |  | 0.7 | (-0.8, 2.1) | 0.30 |
| Cog Fails Distractibility | 9.3 | 1.0 |  |  | 0.4 | (-1.2, 2.0) | 0.60 |
| Cog Fails false Triggering | 5.8 | 0.8 |  |  | 1.3 | (-0.2, 2.9) | 0.09 |
| FACT-G |  |  |  |  |  |  |  |
| Total score | 80.0 | 2.9 | -0.7 | (-5.8, 4.3) | 0.5 | (-4.6, 5.7) | 0.88 |
| Physical wellbeing | 21.2 | 1.2 | -1.8 | (-4.4, 0.7) | -0.9 | (-3.5, 1.7) | 0.37 |
| Social wellbeing | 23.1 | 0.9 | -0.1 | (-1.1, 1.0) | 0.1 | (-0.9, 1.1) | 1.00 |
| Emotional wellbeing | 16.4 | 0.8 | 2.4 | (0.8, 4.0) | 2.8 | (1.2, 4.4) | 0.002 |
| Functional wellbeing | 19.5 | 1.1 | -1.3 | (-3.3, 0.7) | -1.6 | (-3.6, 0.4) | 0.25 |
| FACIT-Fatigue |  |  |  |  |  |  |  |
| Fatigue Total score | 37.3 | 2.3 | -3.5 | (-7.3, 0.4) | -5.4 | (-9.3, -1.5) | 0.03 |
| PROMIS Emotional Distress scales |  |  |  |  |  |  |  |
| Depression 8b | 51.5 | 1.7 | -1.2 | (-4.1, 1.7) | -2.6 | (-5.5, 0.3) | 0.21 |
| Anxiety 7a | 55.7 | 1.8 | -6.6 | (-9.7, -3.6) | -7.6 | (-10.8, -4.5) | <0.001 |

Notes:

chg: mean change from pre-chemotherapy (interpretation of M chg for measures described below); CI: confidence interval; p-value: for global test (F-test) of differences across time, statistically significant results in **bold** for emphasis.

Interpretation of M chg: for the EORTC Cognitive functioning scale Total score, FACT-Cog scales, Cognitive Failures Questionnaire scales, and PROMIS Emotional Distress measures, a positive M chg reflects deterioration, and a negative M chg reflects improvement; for the FACT-G domain and total scores, and FACIT-F Total score, a positive M chg reflects improvement, and a negative M chg reflects deterioration

### Appendix B: Linear mixed model results for neuropsychological tests

| Test/scale | Pre-chemotherapy | |  | Mid-chemotherapy chg | |  | 6-8 weeks post-chemotherapy chg | | | p-value | |
| --- | --- | --- | --- | --- | --- | --- | --- | --- | --- | --- | --- |
|  | est. | se |  | est. | 95% CI |  | est. | 95% CI | |  |  |
| Hopkins Verbal Learning Test |  |  |  |  |  |  |  |  |  | |  |
| Total Recall | 40.8 | 1.9 |  | 4.1 | (0.3, 7.8) |  | 5.2 | (1.4, 9.0) | **0.02** | |  |
| Delayed Recall | 38.1 | 2.2 |  | 3.5 | (-1.0, 8.1) |  | 3.7 | (-0.1, 8.3) | 0.20 | |  |
| Retention | 41.8 | 2.4 |  | 1.4 | (-4.6, 7.4) |  | 0.2 | (-5.9, 6.2) | 0.88 | |  |
| Recognition/Discrimination | 46.7 | 2.2 |  | -7.4 | (-12.0, -2.8) |  | 1.5 | (-3.2, 6.1) | **<0.001** | |  |
| Controlled Oral Word Association Test |  |  |  |  |  |  |  |  |  | |  |
| Total Letter Fluency | 42.3 | 2.1 |  | 5.9 | (2.5, 9.4) |  | 3.6 | (0.1, 7.2) | **0.004** | |  |
| Category Fluency | 46.5 | 2.0 |  | -0.7 | (-3.6, 2.8) |  | -0.1 | (-3.0, 2.9) | 0.88 | |  |
| Total Written Fluency | 44.5 | 2.5 |  | 2.2 | (-0.9, 5.2) |  | 4.1 | (1.0, 7.2) | **0.03** | |  |
| Stroop Colour and Word Test |  |  |  |  |  |  |  |  |  | |  |
| Colour | 43.8 | 1.7 |  | -0.8 | (-2.8, 1.1) |  | -2.3 | (-4.3, -0.3) | 0.08 | |  |
| Word | 43.0 | 1.8 |  | -0.2 | (-3.1, 2.8) |  | -0.7 | (-3.7, 2.3) | 0.88 | |  |
| Word/Colour | 48.2 | 1.7 |  | 0.4 | (-2.1, 2.9) |  | 0.1 | (-2.4, 2.7) | 0.95 | |  |
| Inference Colour/Word | 46.8 | 1.6 |  | 1.4 | (-1.1, 3.9) |  | 1.7 | (-0.8, 4.2) | 0.36 | |  |
| Trail Making Test |  |  |  |  |  |  |  |  |  | |  |
| A score | 45.8 | 1.7 |  | 1.8 | (-1.0, 4.6) |  | 3.0 | (0.1, 5.9) | 0.12 | |  |
| B score | 46.6 | 2.5 |  | 2.4 | (-1.5, 6.3) |  | 0.7 | (-3.2, 4.7) | 0.46 | |  |
| Digit Span Wechsler Adult Intelligence Scale |  |  |  |  |  |  |  |  |  | |  |
| Digit Span Total | 48.2 | 1.6 |  | 1.8 | (-0.7, 4.3) |  | 1.2 | (-1.3, 3.8) | 0.34 | |  |

Notes:

chg: mean change from pre-chemotherapy; CI: confidence interval; p-value: for global test (F-test) of differences across time, statistically significant results in **bold** for emphasis.

Interpretation of M chg: for all measures, a positive M chg reflects an improvement in cognitive performance, and a negative M chg reflects a deterioration in cognitive performance.

*Appendix C: Descriptive statistics for blood cell-based inflammatory markers, pre-chemotherapy scores and mean changes at follow-up assessments*

| Inflammatory marker | Baseline | | | Mid-chemotherapy chg | | | | | 6-8 weeks post-chemotherapy chg | | | | |
| --- | --- | --- | --- | --- | --- | --- | --- | --- | --- | --- | --- | --- | --- |
|  | n | M | SD | n | M | SD | M diff (95% CI) | ES | n | M | SD | M diff (95% CI) | ES |
| NLR | 30 | 3.2 | 2.5 | 30 | 2.8 | 2.4 | 0.7 (-0.9, 1.7) | 0.18 | 29 | 4.3 | 4.5 | 1.0 (-3.8, 0.3) | *0.40* |
| PLR | 30 | 218.6 | 168.4 | 30 | 321.4 | 220.4 | -102.6 (-181.2, -18.1) | *0.61* | 29 | 277.0 | 200.4 | -64.4 (-125.5, 3.6) | *0.35* |
| SII | 30 | 1154.0 | 1293.2 | 30 | 902.4 | 888.6 | 253.5 (-226.1, 903.7) | 0.19 | 29 | 993.2 | 789.0 | 137.4 (-287.3, 741.8) | 0.12 |

Notes:

chg: mean change from pre-chemotherapy; CI: confidence interval; ES: Kazis effect size (interpretation of mean changes: 0.2, small; 0.5, medium; and 0.8, large), medium-sized changes are *italicised* and large-sized changes are in **bold** for emphasis; NLR, neutrophil to lymphocyte ratio; PLR, platelet to lymphocyte ratio; and SII, systemic immune-inflammatory index. Higher blood-cell inflammatory markers reflecting lower cognitive performance.
